# Systemic decoupling of trait coordination: network-based identification of multi-stress resilient maize ideotypes

**DOI:** 10.3389/fpls.2026.1863221

**Published:** 2026-07-08

**Authors:** Huaijun Tang, Lei Zhang, Yi Ren, Xiaoqing Xie, Yejian Wang, Xiaodong Wang, Shengqi Gao, Li Ma, Tianyu Wang, Cheng Liu

**Affiliations:** 1 Institute of Crops Research, Academy of Agricultural Sciences of Xinjiang Uyghur Autonomous Region, Urumqi, China; 2 College of Agronomy, Xinjiang Agricultural University, Urumqi, China; 3 Institute of Crop Sciences, Chinese Academy of Agricultural Sciences, Beijing, China

**Keywords:** abiotic stress, drought tolerance, high-yielding varieties, low nitrogen tolerance, maize, nitrogen use efficiency

## Abstract

The agricultural sector faces significant challenges in maintaining crop productivity under environmental stressors such as drought and low nitrogen (LN) availability. Maize (Zea mays L.), a globally important cereal crop, is particularly vulnerable to these adverse conditions. We evaluated morphological data of 21 maize varieties under drought and low nitrogen. Results showed that vegetative traits showed moderate plasticity, while reproductive synchrony was the primary driver of yield stability. Drought stress led to a ~4-fold expansion of the Anthesis-Silking Interval (ASI), increasing from 0.14 to 0.55 days, which contributed to a 27% reduction in grain yield per plant (GYP). The network analysis showed that under drought, traits became fundamentally decoupled. In well-watered conditions, growth and yield formed a highly coordinated hub, but under water deficit the network fragmented — with tip blanking (r = –0.44) and empty kernel rate (r = –0.37) emerging as the key negative regulators. Conversely, the germplasm showed resilience to low nitrogen, with grain yield per plant (GYP) showing no significant difference from control (215 ± 19.3 g vs. 218 ± 21.5 g; P > 0.05). Under low nitrogen, kernel length (−2.1%, P < 0.05) and hundred-kernel weight (−3.2%, P < 0.05) decreased significantly, but total kernels per ear increased by 7.1% (P < 0.05), fully buffering yield through a sink-protected compensation strategy. Utilizing the Stress Tolerance Index (STI), we identified Xinyu 108, Ruipu 909, and Weike 702 as elite, multi-stress resilient varieties, highlighting their potential for cultivation in resource-limited environments. This research offers valuable insights for breeding programs focused on developing resilient maize varieties.

## 1 Introduction

Maize (*Zea mays* L.) is an important crop, valued not only as a cereal but also as a source of forage, and energy. Its production impacts a substantial portion of the global population (Ahmed et al., 2023; Zia et al., 2023; Sabir et al., 2024). As a C_4_ plant, maize exhibits high photosynthetic efficiency and achieves substantial biomass under an adequate nitrogen (N) supply (Gheith et al., 2022). Cereal crops such as maize are highly susceptible to drought, necessitating significant efforts to develop strategies for improving their tolerance to water deficit stress (WDS) in an era of increasingly frequent extreme weather events (Ran et al., 2023). Since the Green Revolution, farmers have maximized N fertilization to boost crop yields (Żarski and Kuśmierek-Tomaszewska, 2023). Large applications of nitrogen (N) fertilizer are essential for maize development and grain yield (Zhang et al., 2023). However, excessive use since the 2000s has resulted in various environmental issues (Govindasamy et al., 2023; Guo et al., 2023). Excess use of N fertilizer damages the ecological environment, causing phenomena like algal blooms in lakes and red tides in estuaries, and increase NO and NH_3_ emissions from farmlands (He et al., 2023; Feng et al., 2024). The waste of N fertilizer results from the over application to maize plants with low nitrogen use efficiency (NUE), which reflects the ratio of grain yield to nitrogen supplied and encompasses both nitrogen uptake efficiency (NUpE) and nitrogen utilization efficiency (NUtE) (Liu et al., 2023; Dong et al., 2024). While improving NUE is agronomically important, it is distinct from breeding for low-nitrogen (LN) tolerance, which specifically targets the ability to maintain productivity under nitrogen-limiting conditions. This can be achieved by crossing LN-tolerant inbred lines of maize. Breeding LN tolerant varieties could maintain grain yield while reducing the required amount of N fertilizer. The need to screen for LN tolerant cultivars has led scholars to identify reliable indices for selecting LN tolerant phenotypes (Gheith et al., 2022; García-Gutiérrez et al., 2024). Researchers commonly use the relative grain yield of varieties under both LN stress and optimal conditions as a primary selection criterion for identifying desirable phenotypes (Gelaw et al., 2023). Screening indices are mathematical expressions that evaluate plant performance under stress and normal conditions. Various screening indices reflecting stress influence have been proposed based on yield differences across conditions (Ahmad et al., 2022). Moreover, drought stress exacerbates nitrogen limitation by reducing N mineralization in the soil and impairing the transport of nitrogen from roots to shoots, impairing its uptake and utilization in plants (Maywald et al., 2022; Riache et al., 2022). In maize, nitrogen nutrition plays a critical role in modulating plant responses to water deficit stress. For example, an early study by Bennett et al. (1989) found that field-grown maize withstands drought conditions better when grown under low nitrogen levels, emphasizing the importance of proper timing between irrigation and nitrogen application for optimal performance under drought (Bennett et al., 1989). Additionally, Ding et al., 2022 demonstrated that the form of nitrogen (NO^3-^ or NH^4+^) differentially affects plant responses to drought stress, providing valuable insights into the role of nitrogen nutrition in drought resilience (Ding et al., 2022).

Traditional approaches for evaluating multiple stress tolerance rely on pairwise correlation analysis or principal component analysis (PCA) to reduce trait dimensionality (He et al., 2020). However, these methods have critical limitations for understanding complex stress responses. Correlation analysis captures only bivariate linear relationships and fails to reveal higher-order interactions among traits. In contrast, network-based approaches model traits as nodes and their statistical dependencies as edges, enabling the identification of ‘hub’ traits that coordinate stress responses, detection of modular trait clusters that represent functionally integrated response strategies, and quantification of network topological properties (Rao et al., 2023; Medeiros et al., 2025). Applying network-based analysis to LN tolerance screening allows identification of trait modules that coordinate nitrogen-efficient phenotypes, moving beyond single-trait selection indices. Based on the literature reviewed above, we formulated the following testable hypotheses: Drought and low-nitrogen stresses differentially remodel trait network architecture; Drought-tolerant and low-nitrogen-tolerant varieties exhibit distinct network topologies compared to sensitive varieties and Varieties that combine both drought resistance and low-nitrogen tolerance possess unique network configurations.

Despite the progress summarized above, several critical questions remain unresolved. First, it is unknown whether drought and low-nitrogen stresses induce convergent or divergent restructuring of trait network architecture in maize—specifically, whether the same traits serve as network hubs under both stresses or whether each stress recruits distinct coordination modules. Second, the extent to which drought and low-nitrogen tolerance share common phenotypic ‘backbones’ versus independent adaptive pathways has not been systematically investigated at the network level, leaving breeders uncertain whether selection for one stress tolerance will inadvertently enhance or compromise tolerance to the other. Third, while screening indices are widely used for single-stress evaluation, their integration with network topology metrics to identify varieties with robust multi-stress resilience remains unexplored (Rafiq et al., 2024). The present study addresses these gaps by exploring the connection between drought resistance and LN tolerance of maize, and identification of varieties that have both properties and are able to produce optimum yield under drought and LN. The selection of such varieties of maize that are both drought-resistant and low-nitrogen tolerant allows reducing water usage and nitrogen fertilizers, minimizing the negative impact on the environment of agriculture. Rational irrigation and fertilization combined with stress-tolerance properties of maize may enable establishing a sustainable cultivation system. In short, this comprehensive strategy seeks to attain drought tolerance, water conservation, less fertilizers use, efficient resource utilization, increased yield and quality of maize, as well as promoting the green, efficient, and sustainable development of maize production in hot and dry regions.

## 2 Materials and methods

### 2.1 Maize varieties

Twenty-one commercial maize hybrids currently cultivated in the Ili Valley, Xinjiang, China, were selected based on local agricultural extension recommendations and area coverage. These included KWS-series hybrids: KWS9384 (V2), KWS3376 (V4), and KWS2564 (V8) *(*Tang et al., 2024*)*; Denghai-series: Denghai 550 (V12) and Denghai 119 (V17); and regionally popular varieties such as Heyu 187 (V1), Huamei No. 1 (V3), Ruipu 909 (V5), Xinyu 108 (V10), M751 (V6), Wugu 568 (V7), Huaxi 703 (V9), Gaoke 309 (V11), Weike 702 (V13), Jinli 1702 (V14), Jinli 1982 (V15), Longxing 1 (V16), Zheng Pinyu 608 (V18), Jiu Shenghe 3704 (V19), Lihe 869 (V20), and Lihe 879 (V21) *(*Du et al., 2025*)*. These 21 hybrids represent the major genetic backgrounds commercially cultivated in the Ili Valley, encompassing diverse heterotic groups to capture broad adaptive variation. Variety selection was based on area coverage and prior reports of variable stress performance. Several of these varieties have been previously evaluated for abiotic stress tolerance in Xinjiang and low-temperature emergence *(*Tang et al., 2024*)*.

### 2.2 Experimental design

This study was carried out on the Anning Canal Experimental Field belonging to the Xinjiang Academy of Agricultural Sciences, located at 87.49°E longitude, 43.95°N latitude, and 590 m altitude (Tang et al., 2024). The climate of the area is arid and continental. In the maize cultivation period, the average daily temperature fluctuated between 10 °C and 35 °C. Rainfall was low, from 50 to 70 mm, and evaporation was high, from 1500 to 1700 mm, leading to arid conditions both in the atmosphere and in the soil. The fertility of the soil was moderate to high, and there were flat terrains on the experimental land, which ensured that water and nutrients would be distributed evenly. A sequential planting method was used, where each planting row was three meters long. The plant spacing was 0.25 meters, row spacing 0.55 meters, and observation tracks were spaced 0.8 meters apart. The experiment was laid out in a randomized complete block design (RCBD) with three blocks (replications). Randomization was performed using a random number generator in R (v4.3.0) prior to field layout. Treatments were randomly assigned to plots within each block. Each plot consisted of four rows, each 3 m in length, with 0.25 m plant spacing and 0.55 m row spacing. The net harvested area per plot was the central two rows (excluding border rows) to minimize edge effects. The total number of plants per plot was approximately 48 plants (4 rows × 3 m/0.25 m spacing = 48 plants), with ~24 plants in the harvested area. The total number of experimental units was 27 plots (3 treatments × 3 blocks × 3 replications). To minimize potential cross-contamination between experimental units, a 5-meter isolation buffer was maintained around all plots, and a 1-meter alley separated adjacent blocks (Shi et al., 2022). The experiment included three treatments: a normal watering and nitrogen application control plot, a low-nitrogen stress treatment plot, and a drought stress treatment plot (Li et al., 2025b). Each treatment was replicated three times and consisted of four rows. All field management practices for the three treatment plots were carried out on the same day. The application of urea along with the irrigation water was carried out each time. The diammonium phosphate was employed for base fertilization. Apart from the two stressed conditions being imposed by the experiment, there were no other stress factors, and all agricultural operations were done according to standard practice.

### 2.3 Experimental site management

Before sowing in the experimental fields, 300 kg/ha of diammonium phosphate (18% N, 46% P_2_O_5_) and 75 kg/ha of urea (46% N) were uniformly applied as base fertilizer across all plots (Zhang et al., 2017). To improve seedling survival and cut down on bare patches in the experimental plots, we went with plastic film mulching combined with dry sowing with wet irrigation (Xu et al., 2015). Sowing was performed into dry ground on May 17, 2023. Watering was held for next two days. The next day 75 mm of irrigation was done at once. Seedling emergence was observed on May 26, achieving a high emergence rate of 98%. Seedlings were thinned on May 29, and final establishment was completed by June 7. During the seedling stage, we sprayed a mix of pyrozine and nitrate once the crop hit the three-leaf stage, and we ended up making three separate applications to keep cutworms under control. Cultivation occurred twice, and ditches were dug once. Weeding was carried out twice. On June 29, 225 kg/ha of urea was top-dressed along with jointing water in the control and drought treatment plots only, supplying an additional 103.5 kg N/ha. The low-nitrogen treatment received no top-dressed urea at jointing, resulting in a total N application of 34.5 kg N/ha (base fertilizer only) versus 138 kg N/ha in control and drought treatments. On August 4, spider mites were controlled. Each field management measure was completed on the same day, and planting and management were strictly conducted according to the trial plan. Before harvesting the test materials, traits such as growth period, plant height, ear height, plant number, ear number, and yield were investigated.

### 2.4 Water stress treatment

Both the normal irrigation control and the drought stress treatment received sufficient water after sowing to ensure uniform seedling emergence. For the remaining six irrigation events, the drought stress treatment received 50% of the irrigation amount applied in the normal irrigation treatment. During the maize growing season from May to September, the total rainfall was 40 mm. Over the entire growth period, the total irrigation amount for the drought stress treatment was 60.17% of that in the control treatment (**Table 1**). The low-nitrogen treatment received the same amount of irrigation and frequency as the control treatment. The irrigation stages and volume are shown in **Table 1**.

**Table 1:** Irrigation treatment design table.

Treatments	Irrigation (mm)								
	Sowing period	Jointing stage	Whorl stage* (V8-V10)	Tasseling & flowering period	Early grouting	Middle grouting	Later grouting	Full seasonal rainfall	Total
Control (Normal water)	75	75	75	75	75	75	75	40	565
Low Nitrogen	75	75	75	75	75	75	75	40	565
Drought Treatment	75	37.5	37.5	37.5	37.5	37.5	37.5	40	340

*Approximately 8–10 visible leaf collars.

### 2.5 Indicator calculation

Anthesis was recorded when 50% of plants in a plot had begun shedding pollen from the tassel, and silking was recorded when 50% of plants in a plot had visible silk extrusion (Bolaños and Edmeades, 1996). The plot-level ASI was calculated as the difference between the mean silking date and mean anthesis date of all plants within the harvested area (central two rows) of each plot. ASI was expressed in days. The kernel weight per plant and the weight of one hundred kernel are adjusted to a standard moisture content of 14%. The Stress Tolerance Index (STI) was calculated to identify superior varieties, defined as:


$$STI=\frac{\left(Yp\text{ X }Ys\right)}{(\bar{Y}p)2}$$

where *Yp* is the yield under non-stress conditions (Water/Control), *Ys* is the yield under stress (Drought/Nitrogen), and $\bar{Y}$*p* is the grand mean yield across all varieties under non-stress conditions (Azrai et al., 2025).

### 2.6 Statistical correlation and network topology analysis

To evaluate the systemic coordination between phenological, morphological, and productivity traits, pairwise relationship analyses were conducted across all three experimental environments (Control, Low Nitrogen, and Drought). The correlation was carried out using R v4.3.0. Pearson correlation coefficients was calculated using the `cor()` function and `pairwise.complete.obs` package was used to create master file (Schafer and Graham, 2002). Three different matrixes for control, drought and low nitrogen were visualize using the `ggplot2` and `reshape2` packages.

Significance levels for the correlations were determined using a two-tailed Student’s t-test, with an alpha level of P < 0.05 set as the threshold for maintaining edges in the subsequent network analysis (Benjamini and Hochberg, 1995). Nodes and network were generated based on significant Pearson correlation coefficients. The network layout was optimized using the Fruchterman-Reingold force-directed algorithm (Fruchterman and Reingold, 1991). All network graphs were created using the `tidygraph` and `ggraph` packages in VS Code (Wickham, 2016).

### 2.7 Data processing

The analysis environment was managed in environment managed by the `Pixi` package. Data cleaning, outlier detection, and reshaping were performed using the `tidyverse` package (v2.0.0) (Wickham et al., 2019). The average value for each trait was calculated based on three biological replicates per treatment group (Control, Low Nitrogen, Drought). Normality of data distribution was assessed using the `Shapiro-Wilk` test. To evaluate the impact of environmental stress on phenotypic expression, analysis of variance (ANOVA) was conducted (**Supplementary Tables 1**, **2**). Significant differences between treatments and varieties were visualized using distribution-aware plotting techniques.

## 3 Results

### 3.1 Morphological response of maize varieties under different treatments

The recorded data indicate that various nitrogen treatments, drought conditions, and maize varieties significantly affect traits such as days to silking, ASI (Anthesis Silking Interval), plant height, ear height, single ear weight, number of row, ear length, ear area, bald tip, hole ratio, kernel width, kernel thickness, kernel length, hundred kernel weight and kernel weight per plant, as presented in **Table 2**. These traits are primarily influenced by the interaction between genetic and environmental factors. However, the treatments do not significantly affect days to tasseling, days to anthesis and grain row number. The interaction between treatments and varieties did not have a significant effect on days to tasseling, days to silking, plant height, ear height, kernel row number, ear diameter, bald tip length, or kernel thickness. However, it significantly influenced days to anthesis, single ear weight, number of kernels per row, ear length, ear area hole ratio, kernel width, kernel length, hundred-kernel weight, and kernel weight per plant, as shown in **Table 2**. These significantly affected traits are primarily governed by genetic factors and are less influenced by environmental conditions.

**Table 2:** Analysis of variance (ANOVA) for morphological and yield-related traits in maize under different treatments and varieties.

Parameters	Source of variation			
	Treatment (T)	Varieties (V)	T x V	Error
	2	20	40	126
Days to Tasseling	3.0754 ^NS^	32.7821***	1.5212 ^NS^	1.4365
Days to Anthesis	2.1124 ^NS^	31.5124***	2.3152*	1.2249
Days to Silking	8.2116**	30.9942***	2.6839 ^NS^	1.5992
ASI	7.6677***	5.3618***	1.7655**	0.4259
Plant height	16714.8***	3613.0***	49.8 ^NS^	74.90
Ear height	2040.69***	2359.70***	44.86 ^NS^	36.48
Single ear weight	92385.9***	3371.2***	780.9***	283.5
Grain row number	0.39466 ^NS^	9.65648***	0.28921^NS^	0.2880
Number of grain row	383.767***	88.124***	18.748*	10.844
Ear length	4321.77***	404.01***	52.15*	27.81
Ear diameter	138.819***	42.123***	2.137^NS^	1.779
Ear Area	3.277***	10857***	21923*	14615
Bald tip	177.456***	86.790***	5.420 ^NS^	6.654
Hole Ration	192.816***	18.293***	4.947**	2.777
Kernel width	8.5518***	2.6976***	0.1826*	0.1231
Kernel thickness	2.8747**	2.4231***	0.7261^NS^	0.4043
Kernel length	105.688**	4.595***	1.186*	0.5820
Hundred kernel weight	547.545***	55.705***	6.594**	2.134
Kernel weight per plant	74339.6***	2037.4***	564.4**	233.5

***P ≤ 0.001, **P ≤ 0.01, *P ≤ 0.05; *NS*, non-significant.

### 3.2 Effects on flowering traits of maize varieties under different treatments

**Figure 1:**
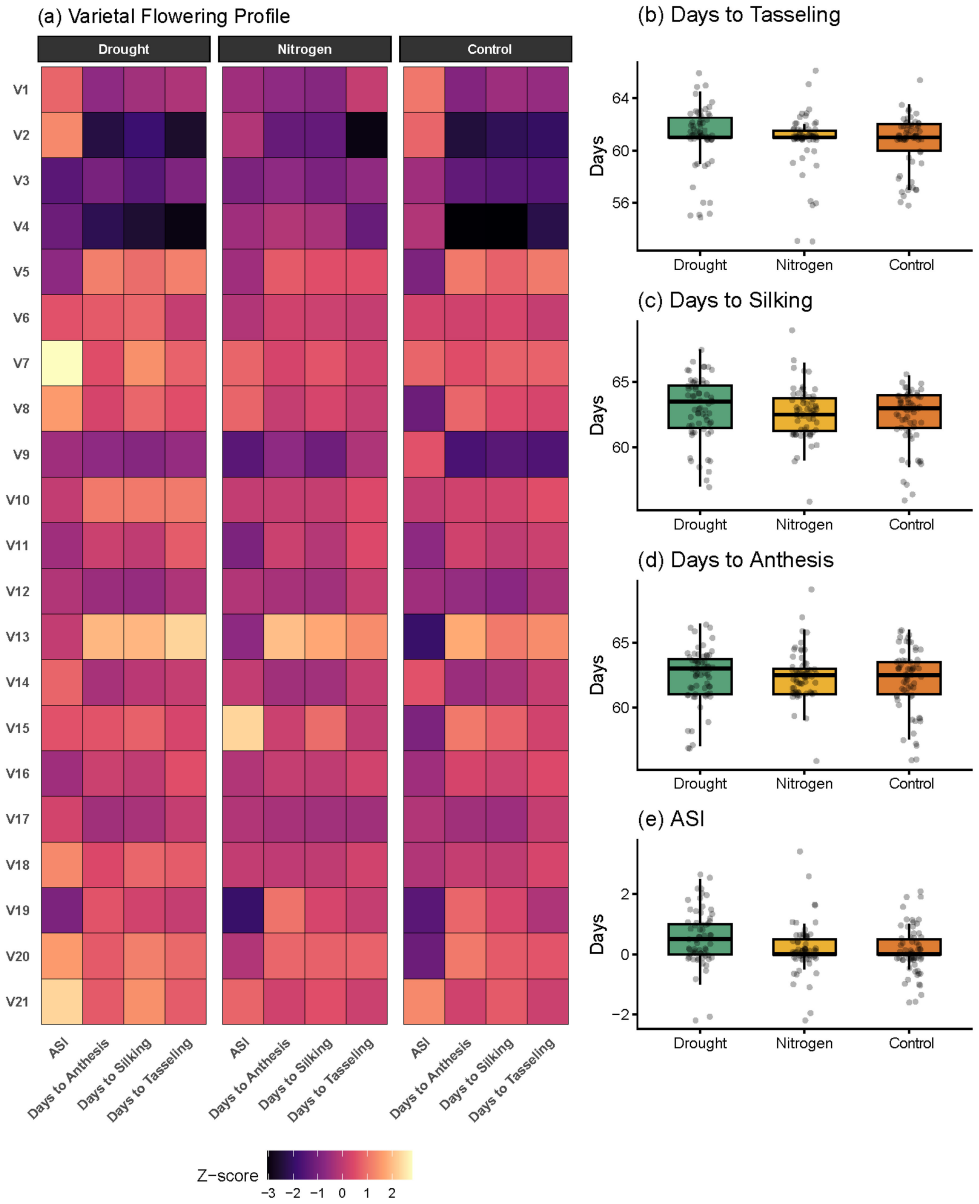
Phenotypic variation in flowering traits under different treatments **(a)** Heatmap of flowering traits across varieties under water (control), low nitrogen, and drought conditions. **(b)** Comparative boxplots for Days to Tasseling (DTT), **(c)** days to silking, **(d)** days to anthesis and **(e)** Anthesis-Silking Interval (ASI).

The detailed analysis of flowering trait was performed using heat map and box plots for different varieties (**Figure 1**). Analysis of flowering traits revealed that while the initiation of flowering was largely conserved across environments, reproductive synchrony was significantly disrupted by water deficit (**Figure 1**). Standardized varietal profiles (**Figure 1a**) identified significant genotypic divergence in this response; notably, varieties V7 (Grain 568) and V8 (KWS2564) exhibited the highest sensitivity, with ASI values reaching 2.5 days under drought. Conversely, the flowering phenology under Low Nitrogen remained comparable to the control. Days to Tasseling (DTT) remained stable, with mean values ranging from 60.6 ± 2.03 days in the control to 61.0 ± 2.39 days under drought stress (**Figure 1b**). As can be seen from **Figure 1a**, the number of days to tasseling and the number of days to anthesis changed slightly under drought and low-nitrogen stress, but their variability decreased. After drought stress, both the number of days to silking and its variability increased significantly, while under nitrogen stress, the change in the number of days silking was not significant (**Figure 1c**). Days to Anthesis (DTA) remained largely unaffected by stress, with mean values of 62.1 ± 2.4 days in control compared to 62.4 ± 2.13 days under drought and 62.5 ± 1.90 days under low nitrogen. Standardized varietal profiles confirmed that while DTA Z-scores remained neutral for most lines, the significant ‘red shift’ in ASI for varieties such as V7 (Grain 568) and V8 (KWS2564) underscores a specific vulnerability in female reproductive development under water deficit. Under drought stress, the anthesis-silking interval (ASI) increased and showed a normal distribution, with significant changes in variability, whereas under nitrogen stress, the change in ASI was relatively small (**Figure 1e**). Heatmap showed that flowering traits like silking and ASI are more significantly affected by drought stress than nitrogen stress. As can be seen from the heatmap (**Figure 1a**), the variety V13 (Weike 702) showed good performance under low nitrogen supply and drought. It had the highest values of tasseling, pollen shedding, and silk emergence among all the tested varieties (**Figure 1a**). At the same time, the lowest results in terms of the indicated factors were obtained by the varieties V4 (KWS3376) and V2 (KWS9384). Thus, both factors affect the main maize characteristics; however, their interaction had a significant effect only on pollen shedding. Among all the tested varieties, the highest tolerance was displayed by the variety V13 (Weike 702), and the worst results – by varieties V4 (KWS3376) and V2 (KWS9384).

### 3.3 Morphological adaptations and plant architecture

**Figure 2:**
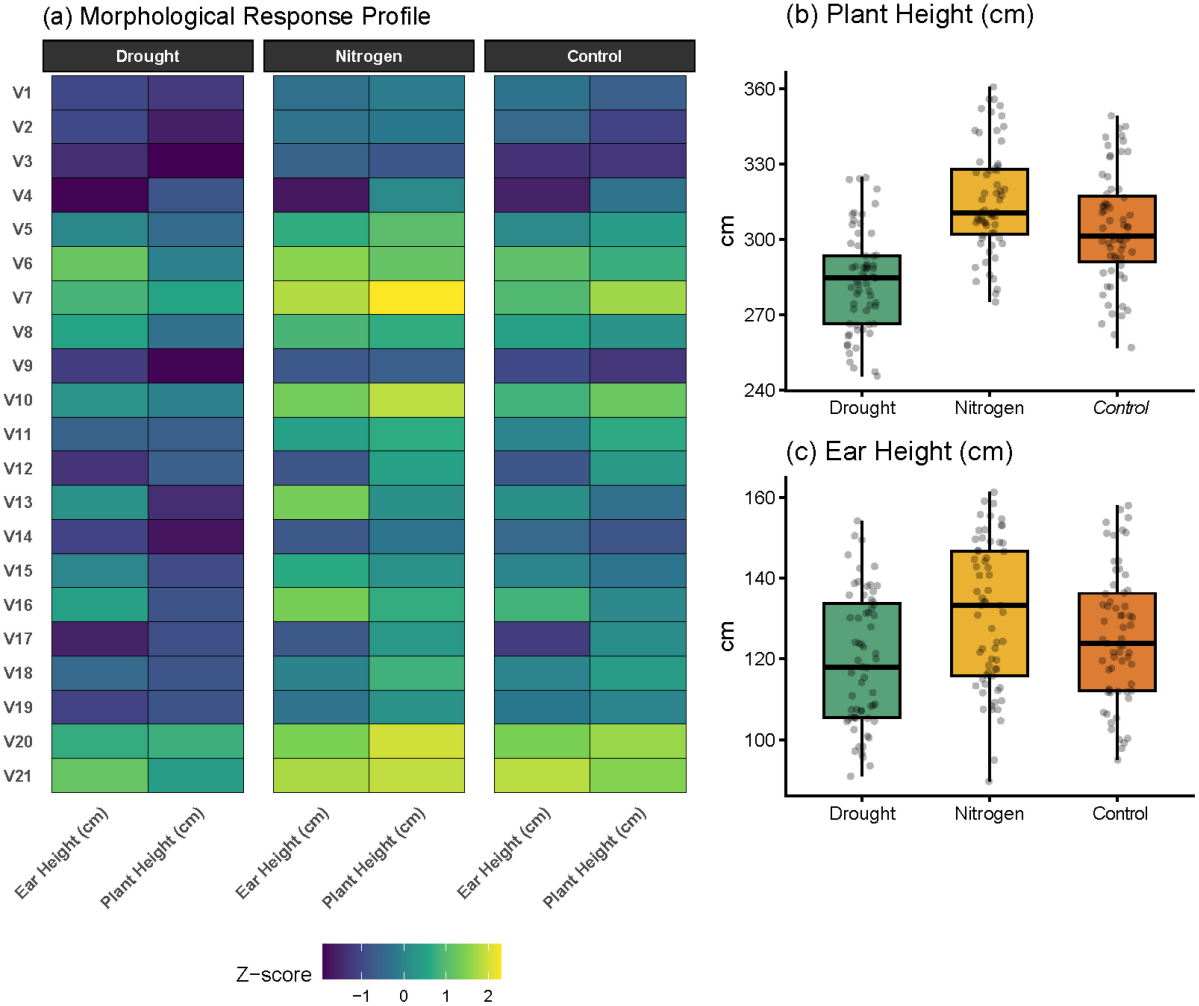
Variation in plant structural traits across environments. **(a)** Standardized heatmap of plant and ear height metrics. **(b)** Boxplot analysis of plant height (cm) and **(c)** ear height (cm).

Maize architectural traits like plant height (Wang et al., 2019) and ear height are critical traits that significantly influence crop yield (Ma et al., 2025). Plant height can be defined as the measure taken between the base of the plant and its highest point of the canopy, while ear height is the vertical distance between the surface and the node where the ear is connected. Both of these characteristics have great importance in assessing the adaptation capacity of the maize plants. Varieties with the superior morphometric measurements were V7 (Grain 568), V20 (Lihe 869), and V21 (Lihe 879) with Z-scores > 1.5 in all environmental scenarios (**Figure 2a**). The concurrent reduction in both PH and EH under drought suggests a uniform inhibition of internode elongation. PH decreased from a mean of 302 ± 22.1 cm in control to 281 ± 20.3 cm under drought stress (**Figure 2b**). Similarly, EH was reduced under drought (118 ± 17.5 cm) compared to control conditions (124 ± 15.6 cm) (**Figure 2c**). Under both drought and nitrogen stress conditions, the variability in plant height remained relatively stable, whereas the variability in ear height increased markedly (**Figure 2c**). These results suggest that while both traits are affected by drought and nitrogen stress, ear height is more strongly influenced than plant height. The graphical representation (**Figure 2a**) illustrates that maize varieties V7 (Grain 568), V20 (Lihe 869), and V21 (Lihe 879) exhibited superior performance under low nitrogen treatments combined with drought stress conditions. These varieties demonstrated the highest values for plant height, and ear height compared to all other varieties. In contrast, the lowest values for these traits were observed in varieties V3 (Huamei No. 1) and V9 (West China 703). While both nitrogen treatments and drought conditions significantly influence key maize traits, the interaction effects show a non-significant impact on plant height, and ear height.

### 3.4 Ear morphological traits and yield components

**Figure 3:**
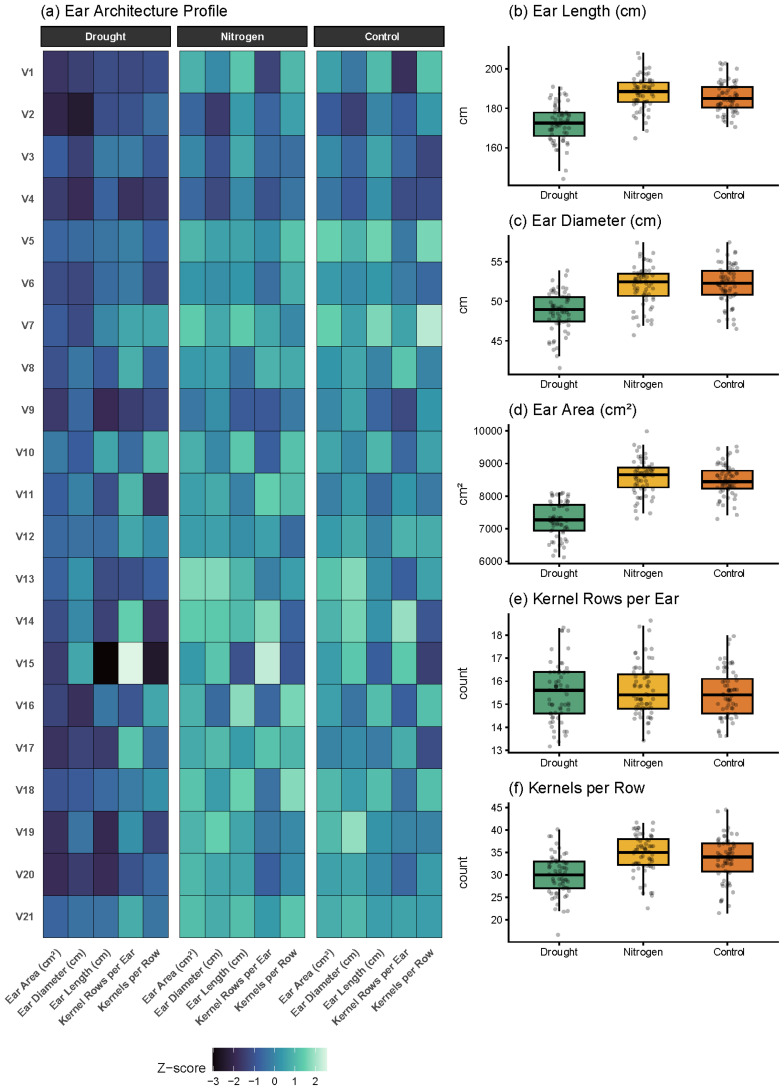
Comparative analysis of maize ear morphology under stress. **(a)** Ear morphology heatmap of ear area, ear diameter, ear length, kernel rows per ear and kernels per row. Boxplots for ear length **(b)**, ear diameter **(c)**, ear area **(d)**, kernel rows per ear **(e)** and kernel per row **(f)**.

As shown in **Figure 3**, significantly restructured ear architecture, primarily through the reduction of longitudinal and radial development (**Figure 3**). Standardized architectural profiles (**Figure 3a**) revealed that varieties V15 (Gold Nugget 1982) and V19 (Jiu Sheng He 3704) were most susceptible to these changes, characterized by intense negative Z-scores across both length and diameter. Variety V16 (Longxing 1) exhibited superior performance under low nitrogen conditions, whereas variety V7 (Grain 568) showed the highest values under control conditions, and variety V10 (Xinyu No. 108) performed best under drought stress. These varieties demonstrated the highest values for ear length, ear diameter, and ear area compared to all other varieties (**Figure 3a**). In contrast, the lowest values for these traits were observed in varieties V2 (KWS9384) and V9 (West China 703) under drought stress conditions. Both nitrogen treatments and drought conditions significantly influence key maize traits, the interaction effects show a non-significant impact on ear length, while significant in ear diameter and ear area of maize. Trait specific heatmap showed that variety V15 (Gold Nugget 1982) had superior performance under combined low-nitrogen and drought stress conditions, while a greater kernel row number was observed in V7 (Grain 568) under control conditions. These varieties demonstrated the highest values for kernel row number and number of rows compared to all other varieties. In contrast, varieties V9 (West China 703) and V11 (Hi-tech 309) exhibited the lowest values for these traits. V15 (Gold Nugget 1982) showed superior performance under combined low-nitrogen and drought stress conditions, while a greater kernel row number was observed in V7 (Grain 568) under control conditions. These varieties demonstrated the highest values for kernel row number and number of rows compared to all other varieties. In contrast, varieties V9 (West China 703) and V11 (Hi-tech 309) exhibited the lowest values for these traits, particularly in kernel row number, while V15 (Gold Nugget 1982) showed the lowest number of rows under drought stress conditions. Varietal performance under drought was highly divergent (**Figure 3a**); genotype V10 (Xinyu No. 108) demonstrated superior resilience, maintaining an ear length (EL) of 188 cm, whereas V15 (Gold Nugget 1982) and V19 (Jiu Sheng He 3704) were identified as highly sensitive, with EL values dropped to 151 cm and 162 cm, respectively. In terms of phenotypic performance, under Low Nitrogen, there was either similar or higher performance compared to control conditions. EL had a significant reduction under drought conditions, where it went down from 186 ± 7.7cm for control to 172 ± 9.61cm (**Figure 3b**). This reduction correlated positively with the reduction in EA from about 14% (**Figure 3d**). There was also a decrease in the diameter of Ear from 52.1cm to 48.6 cm under drought. However, even though this was only a reduction of 6.7%, it should be noted that drought decreased Kernels per Row (KPR). The results indicated that in all three environments, Kernel Rows per Ear were extremely stable, while Kernels per Row decreased from 33.5 ± 4.89 in control to 29.9 ± 4.44 under drought (**Figures 3e, f**).

### 3.5 Kernel productivity and yield stability

**Figure 4:**
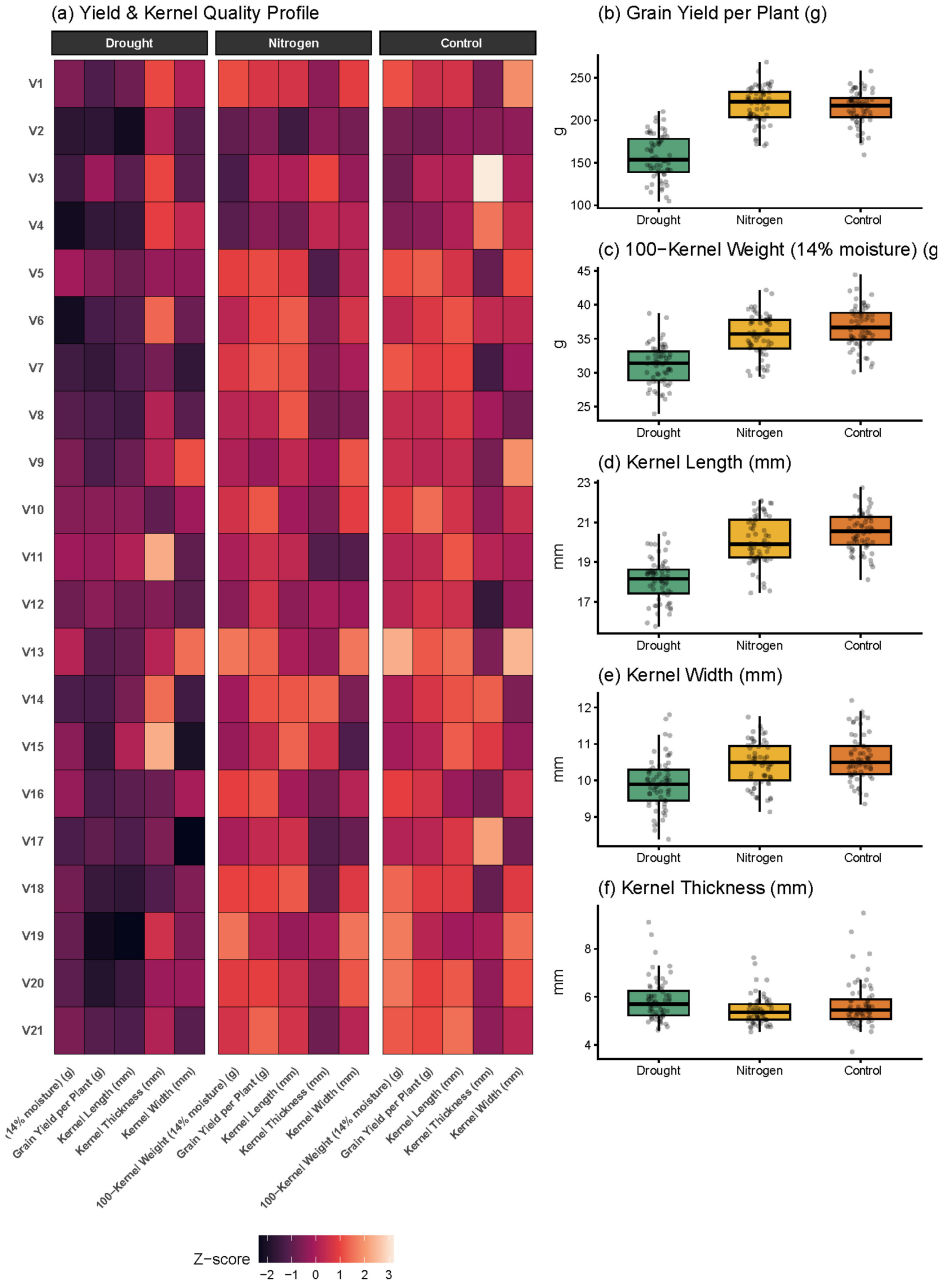
Yield performance of maize varieties. **(a)** Heatmap representing the final productivity levels. **(b)** Boxplots for grain yield per plant (g), 100-kernel weight (HKW) **(c)**, kernel length **(d)**, kernel width **(e)** and kernel thickness **(f)**.

The characteristics of the kernels, including their width, length, and weight per hundred kernels, are vital factors that determine the productivity of maize. These characteristics were evaluated and compared using statistical techniques (**Figure 4a**). The indices for productivity and quality of the kernels proved that moisture stress was the main limiting factor for maize production (**Figure 4**). Varietal screening under drought (**Figure 4a**) identified V3 (Huamei No. 1) and V11 (Hi-tech 309) as the top-performing resilient lines, maintaining yields of 189g and 188g, respectively. Conversely, V19 (Jiu Sheng He 3704) and V20 (Lihe 869) were identified as highly sensitive, with V19 (Jiu Sheng He 3704) producing the lowest mean yield of 122g. Grain Yield per Plant (GYP) exhibited a significant decrease under drought stress, declining by 26.9% from a mean of 215 ± 19.3 g in the control to 157 ± 25.7 g (**Figure 4b**). This yield loss was intrinsically linked to a reduction in kernel size, as evidenced by a 15.2% decrease in 100-Kernel Weight (HKW) (**Figure 4c**) and a reduction in Kernel Length from 20.5 ± 0.96 mm to 18.1 ± 1.04 mm (**Figure 4d**). Kernel traits, such as width, length and hundred-kernel weight, are critical determinants of maize yield as they directly influence the harvest index and overall kernel quality. These traits were measured and analyzed statistically for comparison. As shown in **Figure 4**, after drought stress, kernel width, kernel length, and hundred-kernel weight significantly decreased compared to the control. The variability of these traits after drought stress was smaller than that of the control. The physical quality of the kernel was significantly altered by water deficit (**Figures 4c–f**). Among these morphological changes, the most significant one was related to Kernel Length, which was reduced by roughly 12% (**Figure 4d**). A similar downward trend was observed in Kernel Width, which dropped from 10.6 ± 0.62 mm to 9.91 ± 0.69 mm (**Figure 4e**). Kernel Thickness showed a marginal increase under drought (5.67 ± 0.95 mm to 5.86 ± 0.88 mm), though this change was not statistically significant (P > 0.05) (**Figure 4f**). The concurrent reduction in length and width, paired with the overall 15.2% loss in 100-Kernel Weight (HKW) (**Figure 4c**), indicates that kernel traits are significantly affected by drought stress primarily through longitudinal and radial compression rather than thickness expansion.

“Under low-nitrogen stress, Grain Yield per Plant (GYP) averaged 218 ± 21.5 g, showing no significant difference from the control (215 ± 19.3 g; P > 0.05; **Figure 4b**). This yield stability was underpinned by a significant increase in Total Kernels per Ear (+7.1%, P < 0.05), which compensated for modest but significant reductions in Kernel Length (−2.1%, P < 0.05) and 100-Kernel Weight (−3.2%, P < 0.05). Kernel Width showed minimal changes under nitrogen stress (−1.6%, P > 0.05), and Kernel Thickness also remained statistically unchanged (−4.2%, P > 0.05). The variability of kernel length and hundred-kernel weight increased under nitrogen stress, while kernel thickness variability decreased. These results indicate that low nitrogen stress primarily affects kernel filling (length and weight) rather than kernel set, with the maize germplasm buffering total yield through enhanced kernel number per ear—a sink-protected strategy distinct from the source-driven reproductive failure observed under drought.

### 3.6 Environmental modulation of trait correlations

**Figure 5:**
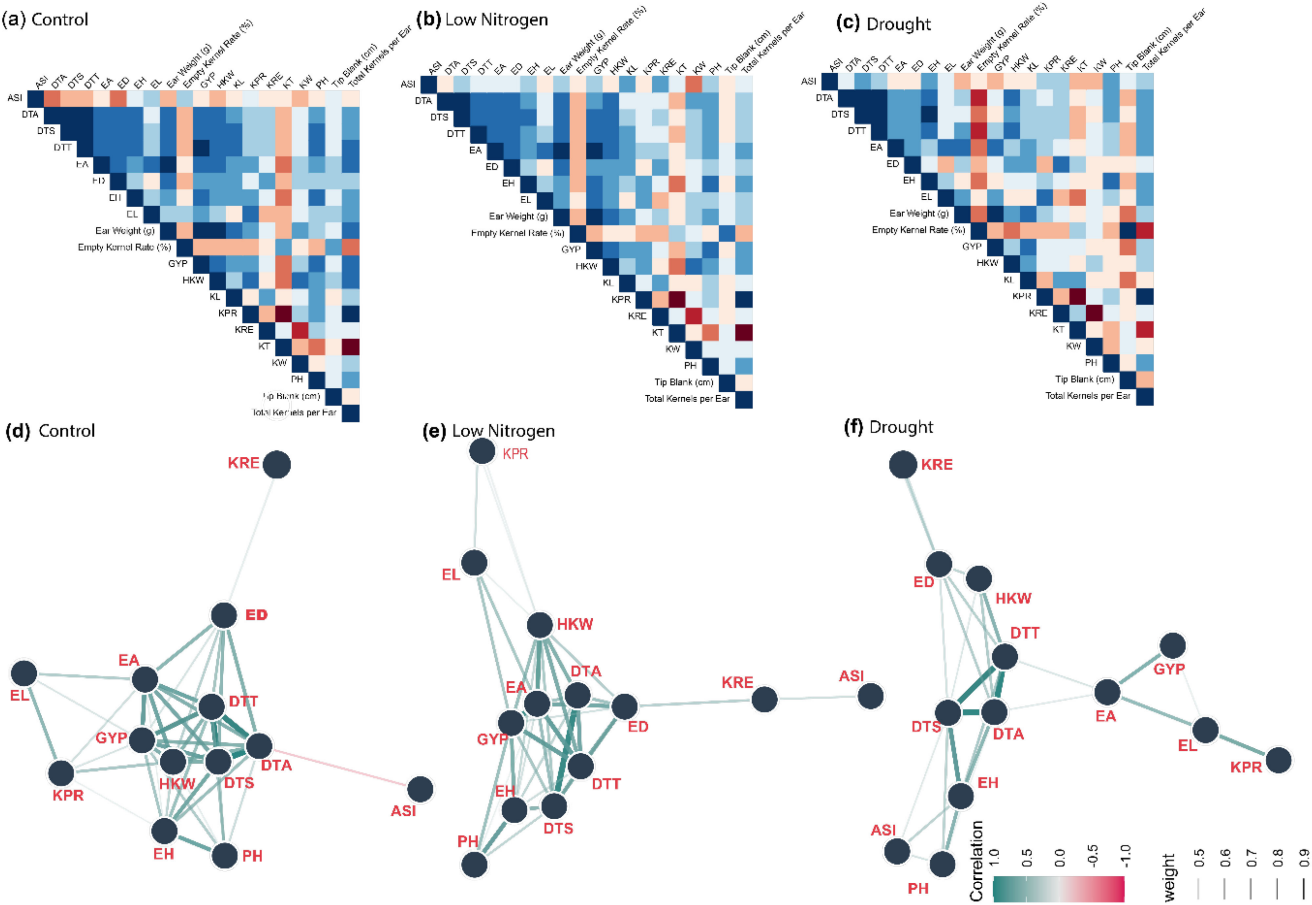
Multi-environmental trait coordination and network analysis of maize varieties. **(a–c)** Triangular correlation matrices under **(a)** water (control), **(b)** low nitrogen, and **(c)** drought conditions. Topographical network diagrams depicting the functional connectivity of the traits under **(d)** water (control), **(e)** low nitrogen, and **(f)** drought. ASI, anthesis-silking interval; DTS, days to silking; DTA, days to anthesis; PH, plant height; EH, ear height; EL, ear length; ED, ear diameter; EA, ear area; GYP, grain yield per plant; HKW, hundred-kernel weight.

To visualize the degree of biological coordination among traits, we employed a correlation and topographical network analysis (**Figure 5**). We integrated phenotypic data into a network-based correlation analysis and presented it in **Figure 5**. The color gradient on the shared legend indicates the strength and direction of the correlation. Under control (**Figures 5a, d**) and low nitrogen (**Figures 5b, e**) conditions, the trait networks exhibited high topological density, with Grain Yield per Plant (GYP) being strongly and positively coordinated with vegetative stature (PH, r > 0.60) and ear morphology (Ear Area, r > 0.79). In contrast, drought stress induced a dramatic reorganization and fragmentation of the trait network (**Figures 5c, f**). Under control conditions (Water), we observed a densely interconnected hub involving ear morphology (EL, ED, EA) and grain yield (GYP). However, under drought stress, the network topology shifted significantly, showing a ‘decoupling’ of flowering traits (ASI) from the yield hub. Yield (GYP), Ear Area (EA), and Plant Height (PH) are all closely clustered in a central hub. In drought Grain Yield (GYP) and Ear Length (EL) are being pulled away from the main flowering cluster (DTS, DTA). Under control conditions (**Figures 5a, d**), the trait network was characterized by high density and strong positive correlations between vegetative stature (PH, EH) and reproductive output (EL, GYP). Under drought condition tip blank, empty kernel rate and ASI have the strongest negative correlations with yield. In Control and Nitrogen, Plant Height is strongly correlated to yield. Across all three treatments, Ear Area remains a top positive driver of yield. This confirms that Ear Area (**Figures 5d–f**) is the core of network architecture. The transformation of the network topology in **Figure 5** proves that yield loss under drought is not a simple linear reduction in growth; it is a fundamental breakdown of the coordination between vegetative stature and reproductive set.

### 3.7 Genotypic ranking for stress resilience

**Figure 6:**
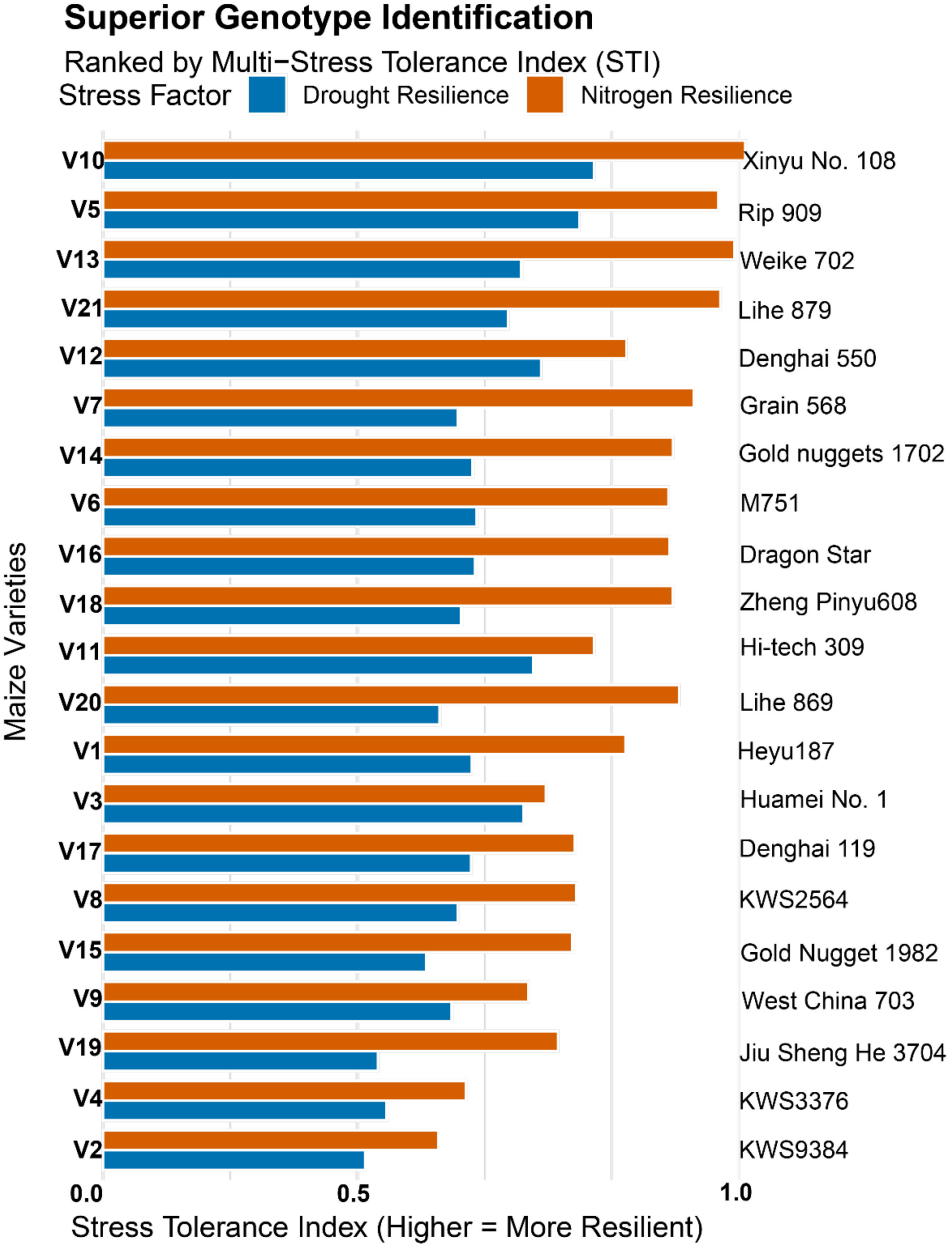
Stress tolerance index (STI) of varieties. Varieties are ranked by their mean resilience across drought (orange) and nitrogen (yellow) stress.

The top tier of the germplasm is dominated by V10 (Xinyu No. 108), V5 (Rip 909), and V13 (Weike 702), which exhibit the highest mean resilience **Figure 6**. V10 (Xinyu No. 108) showed best performance in LN treatment and drought stress. The SCI score was reported highest among the varieties. Such results reveal an impressive capacity for maintaining productivity compared to the mean values. Similar observations were reported in V5 (Rip 909), where it shows good nitrogen resistance than V13 (Weike 702), but the latter exhibits slightly better drought resistance than V5 (Rip 909). V21 (Lihe 879) and V7 (Grain 568) are highly specific for nitrogen stress resistance, as their score was more than 1.1, while their drought stress resistance is moderate. V12 (Denghai 550) provides a much better balance, with its drought tolerance score ranking among the top of the medium range, performing much better than V7 (Grain 568) and V21 (Lihe 879) **Figure 6**. V14 (Gold nuggets 1702), V6 (M751), V16 (Longxing 1), and V18 (Zheng Pinyu608) all show an exceptionally stable performance pattern, scoring almost equally well in their nitrogen tolerance and moderate drought tolerance.

### 3.8 Mechanistic basis of stress resilience

**Figure 7:**
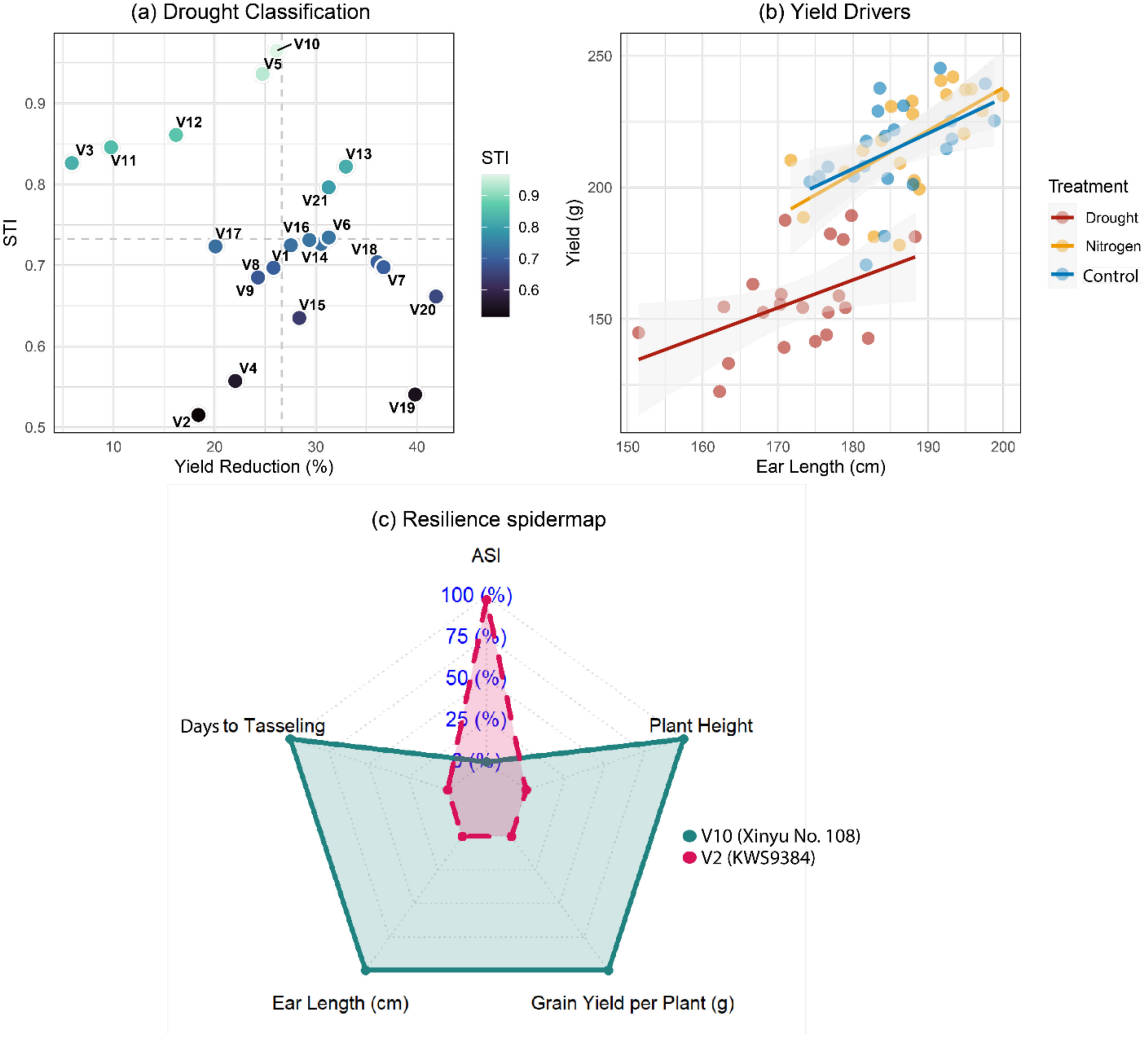
Drought resistant analysis of varieties. **(a)** Variety performance based on yield reduction (%) versus STI. **(b)** Linear regression analysis. **(c)** Spider plot comparing the multi-dimensional functional response of the elite varieties.

In order to find out the physiological mechanism behind stress adaptation, we have generated a multi-dimensional resilience model using phenotypic traits (**Figure 7**). Quadrant analysis of STI against yield penalty percentage identified V10 (Xinyu No. 108) and V5 (Rip 909) as the two most climate-resilient varieties. The two most resilient varieties had an STI value greater than 0.9, maintaining their yields with less than 20% losses. On the other hand, highly sensitive varieties like V2 (KWS9384) and V19 (Jiu Sheng He 3704) had less than 0.6 STI and more than 40% yield losses (**Figure 7A**). Linear regression showed that ear length consistently drove productivity across all environments (p < 0.05). But under drought, the regression intercept was significantly lower than in the control. This suggests that while ear architecture still matters for selection, the plant’s metabolic efficiency in dry conditions holds back its ability to reach full yield potential (**Figure 7B**). A comparative network analysis between the elite variety V10 (Xinyu No. 108) and the sensitive variety V2 (KWS9384) showed just how holistic stress tolerance really is. V10 outperformed V2 across all five axes, most notably in maintaining kernel yield and plant height under limited resources. It also kept a tighter reproductive synchrony (shorter ASI) than V2, which suggests that prioritizing ear development under moisture stress is a hallmark of drought-tolerant maize (**Figure 7C**).

## 4 Discussion

Results from our experiment reveal that stressed maize does not just decrease growth but alters the whole process of resource reallocation and the timing of reproduction. While some morphological features like plant height can be anticipated based on drought levels, the yield drops dramatically when the equilibrium of physiological processes is disturbed. Using phenotypic information together with network modeling, this research provides an effective approach to choosing ideotypes that can maintain sink strength and produce steady yields despite changing climatic conditions. The general consensus is that modern high-yielding crop cultivars are more adaptable to high nitrogen conditions (Riache et al., 2022; Ran et al., 2023). Low nitrogen is considered an abiotic stress for maize plant growth, restricting the expression of their genetic potential during growth and development stages, thereby impacting grain yield performance (Wu et al., 2023). Researchers have typically found that maize accessions are selected appropriately when treated with minimal nitrogen input (Liu et al., 2023). Tasseling timing is crucial for maize yield, and recent studies have examined how different treatments influence this stage. For example, Guo et al. (2021) found that optimized irrigation schedules can advance tasseling and improve overall maize yield by ensuring more consistent moisture availability (Guo et al., 2021). Additionally, highlighted that controlled-release nitrogen fertilizers can synchronize tasseling with other developmental stages, potentially boosting yield outcomes (Kontárová et al., 2022). Demonstrated that certain maize varieties with specific genetic traits show altered tasseling responses to varying nitrogen levels (Sun et al., 2022). Recent research has focused on mitigating issues related to nutrient management. Reported that balanced nutrient management, including phosphorus and potassium, significantly reduced kernel looseness by enhancing kernel integrity and resistance to environmental stress (Hauser, 2020). Stefanov et al. (2020) indicated that adjusting planting density can also affect kernel cohesion and reduce the incidence of loose kernels (Stefanov et al., 2020). Days to silking, which affects pollination efficiency, is another critical trait influenced by various treatments. Precision irrigation and adequate soil moisture during silk elongation can substantially improve silk length and density, leading to better pollination and higher yields (Zizinga et al., 2022; Zhao et al., 2023). Ahmad et al. (2022) showed that different maize varieties exhibit variable responses to drought stress during silk production, with some varieties maintaining better silk quality under stress conditions due to their inherent drought resistance traits (Ferreira et al., 2023; Kim and Lee, 2023).

### 4.1 The dominance of reproductive synchrony over vegetative vigor

The maize yield is often linked to many factors. Flowering is one of the main factors that regulate overall yield in crops. Heatmap and box plots revealed that while days to anthesis remained highly conserved across all three environments, drought stress induced expansion of the ASI. This finding matches the protandry response commonly seen in water-stressed maize where pollen is shed on schedule, but silk growth lags behind due to both physical constraints and metabolic slowdown (Herrero and Johnson, 1980; Li and Xu, 2025). The delay in this process is related to pollination timing and ultimately it affects pollination process. Our study also found that the expansion of the ASI under drought was the primary reason for the increased Empty Kernel Rate (37.5%) and Tip Blanking (44.1%). This clearly evidences that yield loss was not due to photosynthesis, but failure of proper pollination was main reason behind it. Varieties like V10 (Xinyu No. 108) maintained a narrow ASI, enabling successful fertilization, susceptible varieties such as V19 (Jiu Sheng He 3704) and V2 (KWS9384) exhibited high level of asynchrony, leading to severe kernel abortion and reduced Kernels per Row.

### 4.2 Plasticity of morphology under stress conditions

Under drought conditions, plants redirect limited sugars and water away from the development toward survival functions. The core metabolomic functionality governed by antagonistic relationship between growth-promoting and stress-responsive signaling pathways (Kim et al., 2026). Water stress physically slows silk growth and reduces metabolic activity in the ear shoot, while pollen shed stays relatively on schedule (Virág et al., 2026). This phenomenon is linked to disrupting reproductive synchrony (Danilevskaya et al., 2019). Moreover, drought stress triggers abscisic acid (ABA) and ethylene buildup in ovaries, which can trigger embryo abortion even before obvious wilting occurs (Boyer and Westgate, 2004). In the present study, this mechanism was phenotypically manifested as a significant reduction in Kernels per Row and Total Kernels per Ear under drought, while Kernel Rows per Ear remained stable. The preferential loss of kernels along the ear’s longitudinal axis—rather than across rows—indicates that ABA/ethylene-induced abortion targeted developing ovules after row determination but before full kernel set. Furthermore, Kernel Rows per Ear remained remarkably stable across all three environments, Ear Length and Kernels per Row exhibited significant plasticity, particularly under drought. The stability of kernel row number suggests that this trait is determined early in the maize ontogeny. In contrast, the significant reduction in Ear Length (7.1%) and Ear Area (14%) under drought reflects a failure in rachis elongation and subsequent ovary development. Our results show that the drop in 100-Kernel Weight and the collapse in total yield were not merely due to smaller grains but were primarily driven by longitudinal growth of the ear. In a recent study it was reported that ear elongation rates under drought decline to 0.3 cm/day compared to 0.5–0.7 cm/day under well-watered conditions, resulting in ears that are approximately 50% shorter at silking, whereas tassel length is reduced by only 20% under identical stress regimes (Danilevskaya et al., 2019).

### 4.3 Network decoupling and the identification of multi-stress ideotypes

Trait based network analysis has become an important systems-level approach to dissect the intricate relationships among agronomic traits and their collective influence on yield architecture (Wang et al., 2024). Unlike traditional pairwise correlation analyses, network topology captures the emergent properties of trait interactions, revealing how vegetative and reproductive traits synchronize or antagonize under varying environmental regimes (Bertolini et al., 2025). In woody plant communities across an aridity gradient, Medeiros et al. (2025) demonstrated that increasing drought stress simplifies trait network architecture. Similarly, a recent research showed that low light stress in submerged macrophytes significantly reduced trait network connectivity, with increased trait modules responding independently to stress (Rao et al., 2023). Furthermore, in a very relevant research it was demonstrated that nitrogen and phosphorus additions differentially alter multiple functional traits and their associations in desert ephemerals (Tao et al., 2022). In the present study, topological networking of trait networks under varying environments showed that plants work under certain manners where vegetative vigor and reproductive output are tightly synchronized. Specifically, in the Water/Control and Nitrogen networks (**Figures 5d, e**), Plant Height (PH) and Ear Height (EH) maintained strong positive correlations with Grain Yield per Plant (GYP). Previous report also supports the findings. In a recent study it was reported that PH and EH are critical traits in maize and are closely associated with grain yield and mechanized harvesting (Wen et al., 2025). The strong association between the PH-EH-GYP axes, identified above, corresponds with earlier observations indicating that both traits of plant and ear height are associated genetically as well as pleiotropically and have loci responsible for regulation of carbon allocation and structure formation (Wang et al., 2023; Wen et al., 2025). Also, in earlier studies, it was found that increased difference between male and female flowering led to reduced pollination rate and kernel formation, especially during abiotic stress (Li et al., 2025a); similarly our study also supported the findings. We observed that ASI, Tip Blanking, and Empty Kernel Rate proved to be major yield inhibitors.

The conclusions in the study are valid with some limitations like the germplasm panel comprised selective commercial hybrids currently cultivated in the local region and may not represent the full genetic diversity of maize adaptation to drought and low-nitrogen stresses. The regional restriction of the germplasm limits the generalizability of our findings to other agro-ecological zones with different soil types, temperature regimes, and stress intensity profiles (Riaz et al., 2025). The experiment was conducted at a single location over one growing season, precluding assessment of genotype × environment (G×E) interactions that are known to strongly influence maize yield stability under stress. Moreover, we did not examine genotype × environment × management (G×E×M) interactions, such as the effects of planting density, irrigation scheduling, or fertilizer placement on trait expression and network topology.

## 5 Conclusion

The selection of maize varieties that have high yield potential, drought tolerance, and nitrogen tolerance is vital in increasing productivity and achieving food security in areas where there are water shortages and nutrient deficiencies. In our systematic study, we adopted an approach involving the integration of multiple traits and network analysis to determine the genotypes that perform well under stress conditions. We discovered substantial variations in the architecture and systems of the genotypes, whereby drought stress led to a drastic increase in the Anthesis-Silking Interval (ASI), but some genotypes exhibited resistance. These superior varieties supported high yield potential by altering their morphology, specifically Ear Area and Kernel Length—despite water limitations. Similarly, genotypes with low-nitrogen tolerance showed high nutrient-use efficiency, maintaining yield stability despite modest reductions in kernel size. Based on the Stress Tolerance Index (STI) and overall productivity profiles, the maize varieties Xinyu 108 (V10), Ruipu 909 (V5), Weike 702 (V13), Lihe 879 (V21), Longxing 1 (V16), and Zheng Pinyu 608 (V18) are identified as elite drought-resistant and low-nitrogen tolerant types.

## Data Availability

The original contributions presented in the study are included in the article/**Supplementary Material**. Further inquiries can be directed to the corresponding authors.
